# Exopolysaccharides of *Lactobacillus rhamnosus* GG ameliorate *Salmonella typhimurium*-induced intestinal inflammation via the TLR4/NF-κB/MAPK pathway

**DOI:** 10.1186/s40104-023-00830-7

**Published:** 2023-03-06

**Authors:** Jinze Li, Qiuke Li, Qianhui Wu, Nan Gao, Zhihua Wang, Yang Yang, Anshan Shan

**Affiliations:** Institute of Animal Nutrition, Northeast Agricultural University, Harbin, 150030 P. R. China

**Keywords:** Exopolysaccharides, *Lactobacillus rhamnosus*, *Salmonella typhimurium*, TLR4/NF-κB/MAPK

## Abstract

**Background:**

*Salmonella typhimurium* (*S.T*), as an important foodborne bacterial pathogen, can cause diarrhea and gastroenteritis in humans and animals. Numerous studies have confirmed that exopolysaccharides (EPSs) have various biological functions, but the mechanism through which EPSs improve the immunity of animals against the invasion of pathogenic bacteria is unclear. Here, we explored the protective effect of EPSs of *Lactobacillus rhamnosus* GG (LGG) on the *S.T*-infected intestine.

**Methods:**

Mice received adequate food and drinking water for one week before the start of the experiment. After 7 d of prefeeding, 2×10^8^ CFU/mL *S.T* solution and an equivalent volume of saline (control group) were given orally for 1 d. On the fourth day, the mice were treated with 0.5 mg/mL EPSs, 1.0 mg/mL EPSs, 2.0 mg/mL EPSs, or 2.0 mg/mL penicillin for 7 d. Finally, the body and relative organ weight, histological staining, and the levels of antioxidant enzyme activity and inflammatory cytokines were determined.

**Results:**

The *S.T*-infected mice exhibited symptoms of decreased appetite, somnolence, diarrhea and flagging spirit. Treatment with EPSs and penicillin improved the weight loss of the mice, and the high dose of EPSs showed the best therapeutic effect. EPSs significantly ameliorated *S.T*-induced ileal injury in mice. High-dose EPSs were more effective than penicillin for alleviating ileal oxidative damage induced by *S.T*. The mRNA levels of inflammatory cytokines in the ileum of mice showed that the regulatory effects of EPSs on inflammatory cytokines were better than those of penicillin. EPSs could inhibit the expression and activation of key proteins of the TLR4/NF-κB/MAPK pathway and thereby suppress the level of *S.T*-induced ileal inflammation.

**Conclusions:**

EPSs attenuate *S.T*-induced immune responses by inhibiting the expression of key proteins in the TLR4/NF-κB/MAPK signaling pathway. Moreover, EPSs could promote bacterial aggregation into clusters, which may be a potential strategy for reducing the bacterial invasion of intestinal epithelial cells.

## Introduction

Diarrheal illness resulting from various enteropathogenic microorganisms is a considerable threat to human health and the economy in developing countries [1]. *Salmonella* is a common foodborne pathogen that can cause diarrhea outbreaks in humans and animals and even sepsis, meningitis and pneumonia [2]. Rapid and accurate detection of the foodborne pathogen *Salmonella* is important to ensure food safety in the production processes for food such as eggs, poultry and meat products [3]. Common foodborne *Salmonella* pathogens causing food poisoning are *Salmonella typhimurium* (*S.T*), *Salmonella cholera*, and *Salmonella enteritis*, among others [4]. The invasion process of pathogens (such as *S.T*) consists of invasion and colonization, establishment of the pathogen-containing vacuole and spread of the infection to adjacent cellular tissues. This process is controlled by the intestinal epithelium, gut-associated immune system and intestinal microbiota [5]. Bacterial infection of the intestinal tract generally influences the health of the animal and human, thus, in the context of the reducing and prohibiting antibiotics, effective treatments for intestinal diseases caused by bacterial infection and the mitigation of foodborne bacteria caused by damage to the human gastrointestinal tract have become a research orientation focus [6].

In the intestinal tract, abundant probiotic bacteria exist and play essential roles in conferring host health benefits mainly by three general mechanisms: direct inhibition of pathogens and indirect influence on the commensal microbiota, enhancement of the epithelial barrier function via modulation of signaling pathways, and exertion of strain-specific local and systemic effects via modulation of host immune responses [7]. Among probiotic bacteria, *Lactobacillus rhamnosus* GG (LGG, ATCC 53103), which was originally isolated from fecal samples of a healthy human adult [7], may have anti-inflammatory properties that are stimulated nonspecifically by enhancing phagocytic activity and regulating gut microbes [8] and has been used in various commercially available probiotic products under different trademarks [9]. Probiotics are by definition living organisms that must contain an effective quantity of viable bacteria when administered to the host; however, most probiotic preparations, particularly those near the end of their shelf life, also contain potentially significant amounts of dead and damaged microorganisms [10]. As a result, the use of probiotics is quite restricted, and their secondary metabolites, which are known as exopolysaccharides (EPSs), have many beneficial properties. Microbial EPSs applications are frequently employed in fields such as industry (cosmetics, food, textile), health (medicine and pharmaceuticals), and the environment (remediation); and applications related to the advancement of health will become a significant milestone in the coming years [11]. Numerous studies have confirmed that EPSs have various biological functions, which mainly include antitumor activity [12, 13], antibacterial activity, antioxidant activity [14, 15], immunomodulation effects [16] and regulation of the gut microbiota [17]. Our previous research also revealed that the EPSs produced by LGG exhibited effective functions in alleviating the oxidative damage and apoptosis of intestinal porcine epithelial (IPEC-J2) cells and showed great cytocompatibility [18].

Although these studies have confirmed the biological functions of EPSs in vitro, the specific mechanisms through which EPSs exert their functions in vivo are unclear. As a result, we conducted a preliminary analysis of the mechanism of action of EPSs in preventing *S.T* infection and employed EPSs of LGG for the first time in the treatment of *S.T*-induced diarrhea in mice. Here, we found that EPSs isolated and purified from LGG medium alleviated *S.T*-induced intestinal pathological damage and inflammation by maintaining the oxidative/antioxidant balance and modulating the TLR4/NF-κB/MAPK pathway in mice. These finding show that EPSs have potential as a novel medication for the treatment of diarrhea caused by bacteria and intestinal injury.

## Materials and methods

### Microorganism and culture conditions

*Salmonella typhimurium* ATCC 14028 and *Lactobacillus rhamnosus* GG ATCC 53103 were obtained from the Institute of Animal Nutrition, Northeast Agricultural University (Harbin, Heilongjiang, China). Luria-Bertani (LB) medium was used to activate and incubate *S.T* at 37 ℃ in a shaker incubator until the logarithmic growth phase. The bacterial cells were collected by centrifugation at 1000 × *g* for the subsequent experiments. The crude EPSs were separated and purified according to described procedures with slight modifications [18]. In brief, LGG was cultured at 37 ℃ for 36 h in skim milk-modified medium supplemented with 10% glucose. And then heated in a boiling water bath for 10 min to denature the proteins and inactivate the enzymes. Cells and proteins were removed by centrifugation at 10,000 × *g* and 4 ℃ for 15 min. After filtering to obtain the supernatant, trichloroacetic acid was added to a final concentration of 4% (w/v) for 12 h. The supernatant was obtained by centrifugation, 3 times the volume of precooled ethanol was added to the supernatant, and the mixture was incubated at 4 ℃ for 12 h and then centrifuged to obtain the precipitate. The precipitate was dissolved in deionized water, dialyzed using a dialysis bag (molecular weight cutoff of 8000–14,000 Da) for 72 h and then lyophilized in a freeze-dryer. The content of EPSs was analyzed using the phenol sulfuric acid method.

### Animals and experimental design

**Fig. 1:**
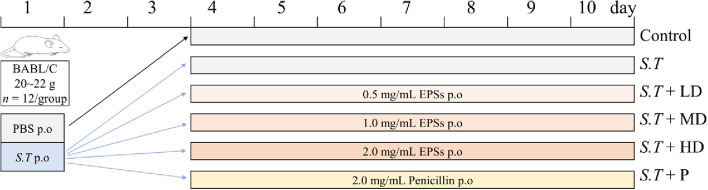
Experimental design. On the first day of the official experiment, 2×10^8^ CFU/mL *S.T* solution and an equivalent volume of PBS (control group) were administered orally for 1 d. On the fourth day, the mice in the *S.T* + LD, *S.T* + MD, *S.T* + HD and *S.T* + P groups were intragastrically administered 0.5 mg/mL EPSs, 1.0 mg/mL EPSs, 2.0 mg/mL EPSs, or 2.0 mg/mL penicillin for 7 d. The mice in the control and *S.T* groups were administered the same amount of PBS

Seventy-two 4-week-old male and female BALB/C mice (20–22 g) were provided by Liao Ning Chang Sheng Biotechnology Co., Ltd., (China), and randomly divided into 6 groups (*n* = 12): control (PBS), *S.T* (*S.T* + PBS), *S.T* + LD (*S.T* + 0.5 mg/mL EPSs), *S.T* + MD (*S.T* + 1.0 mg/mL EPSs), *S.T* + HD (*S.T* + 2.0 mg/mL EPSs) and *S.T* + P (*S.T* + 2.0 mg/mL penicillin) (Fig. 1). The mice were housed in an air-conditioned animal room with an indoor temperature of 23 ± 1 ℃, 40%–60% relative humidity, and 12 h of light daily. The mice received adequate food and drinking water for one week before the start of the experiment. Mouse body weight data were recorded daily throughout the duration of our experiment. At the time of sacrifice, the mice were anesthetized by diethyl ether inhalation, and blood samples were collected retro orbitally. The mice were sacrificed by the cervical dislocation method, and organs were weighed at sacrifice to calculate organ indices as follows: organ index = fresh weight of organs (g)/body live weight (g) × 100%. The collected blood was allowed to stand at room temperature for 30 min and then centrifuged at 1000 × *g* for 20 min, and the serum was collected and stored at −80 ℃ for subsequent experiments. Ileal tissues were then rapidly transferred into 10% (v/v) formaldehyde solution to observe changes in the intestinal morphology, and other parts of the tissues were frozen in liquid nitrogen and stored at −80 ℃ for subsequent experiments.

### Determination of the serum cytokine levels

The expression levels of interleukin-1β (IL-1β), interleukin-2 (IL-2), interleukin-4 (IL-4), interleukin-6 (IL-6) and tumor necrosis factor-α (TNF-α) were measured using ELISA kits from Shanghai Jinma Biotechnology Co., Ltd. (Shanghai, China) according to the manufacturer’s instructions.

### Intestinal histological evaluation

The ileum tissues were collected, fixed with 4% paraformaldehyde and embedded in paraffin. The sections were then stained with H&E, dehydrated with ethyl alcohol and cleared with xylene. The pathological changes in the ileum were observed under an optical microscope (Nikon Eclipse Ci-L, Tokyo, Japan).

### Biochemical assays

The ileal tissue was weighed accurately, and 9 times the volume of saline was added according to the ratio of 1:9 (w:v). After the tissue was cut, homogenate was prepared in an ice water bath and centrifuged for 10 min at 1000 × *g*, and the supernatant was collected for measurement. The levels of malondialdehyde (MDA), hydrogen peroxide (H_2_O_2_), catalase (CAT) and superoxide dismutase (SOD) in the ileum were determined using assay kits (Jiancheng Bioengineering Institute, Nanjing, Jiangsu, China) following the manufacturer’s protocols: the thiobarbituric acid method was used to determine the MDA levels, the spectrophotometry method was used for determination of H_2_O_2_ activity, the visible light method was utilized to measure the CAT levels, and the WST-1 method at 450 nm was used for the assessment of SOD activities. Enzyme activity is expressed as nanomoles per milligram protein.

### Determination of mRNA expression of inflammatory-related genes

To explore whether EPSs of LGG can regulate intestinal immune responses, total RNA of ileum samples was extracted by successively using TRIzol, trichloromethane, isopropanol, diethylpyrocarbonate-ethanol, and diethylpyrocarbonate-water. The integrity of the extracted RNA was evaluated using 1% agarose gels, and their quantity and quality were detected with a Nanodrop spectrophotometer P330 (Implen GmbH, Munich, Germany). cDNA was obtained using the PrimeScript^TM^ RT Reagent Kit (Takara Code: RR047A, Dalian, Liaoning, China). Real-time PCR was performed using TB Green Premix Ex Taq (Takara Code: RR420A, Dalian, Liaoning, China). The primers used in this study are listed in Table 1. The primers were obtained from Shanghai Shenggong Biology Engineering Technology Service, Ltd. (Shanghai, China).

**Table 1:** Primer design and synthesis

**Gene**	**Sequence (5' to 3')**	**Accession number**
*β-actin*	F: GGAGATTACTGCCCTGGCTCCTA	NM_031144.3
	R: GACTCATCGTACTCCTGCTTGCTG	
*TNF-α*	F: CGTCGTAGCAAACCACCAAG	XM_034524601.1
	R: TTGAAGAGAACCTGGGAGTAGACA	
*IL-1β*	F: TCGGCAAAGAAATCAAGATGGC	XM_021194894.2
	R: GTGCAAGTCTCATGAAGTGAGC	
*IL-2*	F: CCAAGCAGGCCACAGAATTG	XM_031376265.1
	R: GCTGACTCATCATCGAATTGGC	
*IL-4*	F: CTTCCAAGGTGCTTCGCATA	NM_021283.2
	R: GATGAATCCAGGCATCGAAA	
*IL-6*	F: ACAGAAGGAGTGGCTAAGGA	NM_031168.2
	R: AGGCATAACGCACTAGGTTT	

### Western blot analysis

The ileal segment was homogenized and treated with RIPA and PMSF for the extraction of total protein. The lysate was centrifuged at 10,000 × *g* and 4 ℃ for 30 min. The protein concentrations were determined using the BCA Protein Assay Kit (Solarbio, Beijing, China). Cellular protein extracts were separated by electrophoresis using a 12% SDS-polyacrylamide gel and electroblotted onto a polyvinylidene fluoride (PVDF) membrane. The PVDF membrane was then blocked with blocking solution (5% nonfat milk powder) for 2 h at 37 ℃. The membrane was incubated with the primary antibody of the protein of interest at 4 ℃ overnight and then subjected to three 10-min washes with Tween 20/Tris-buffered saline (TBST). The membrane was incubated with the HRP-labeled secondary antibody corresponding to the primary antibody for 1 h at 37 ℃ and washed with TBST as described above. Finally, an Electrochemiluminescence Plus Western Blotting Detection System Kit (Beyotime, Shanghai, China) was used to visualize the protein bands according to the manufacturer’s instructions.

### Scanning electron microscopy (SEM)

*S.T* was activated and cultured in LB overnight to a logarithmic period, harvested by centrifugation at 1000 × *g* for 10 min, and diluted to an OD_600_ of 0.2. EPSs were incubated with the bacteria at 37 ℃ for 1 h and harvested by centrifugation. The harvested bacteria were then fixed with 2.5% glutaraldehyde overnight. A graded ethanol series (50%, 70%, 90%, and 100%) was used to continuously dehydrate the bacterial samples for 8 min, and a mixture (v:v = 1:1) of 100% alcohol and tert-butanol was used to dehydrate the bacterial samples for 30 min. The bacterial samples were treated with tert-butanol alone for 1 h. The specimens dried using a critical point dryer were coated and visualized under a field emission scanning electron microscope (Hitachi S-4800, Tokyo, Japan).

### Killing bacterial kinetics

Briefly, *S.T* cells were harvested by concentration at 1000 × *g* for 10 min and washed 3 times with sterile PBS. Different concentrations of EPSs and penicillin (2.0 mg/mL) were then added to 10^5^ CFU/mL *S.T* solution. At each time point, a 10-μL aliquot from treated *S.T* solution, which was serially diluted from 10^−2^ to 10^−7^ in tenfold increments, was placed on Mueller Hinton agar (MHA) and incubated at 37 ℃ overnight.

### Statistical analysis

All the data in the experiments were analyzed by one-way ANOVA using SPSS 22.0 (IBM Inc., Armonk, New York, USA) to determine whether there were significant differences among the groups. The data are expressed as the mean ± SD. The correlations were evaluated by Pearson correlation analysis of the Euclidean distance using GraphPad Prism 9.0 (GraphPad Software, San Diego, CA, USA). *P* < 0.05 was considered to indicate a significant difference.

## Results

### EPSs attenuated the *S.T*-induced weight loss and swelling of organs in mice

**Fig. 2:**
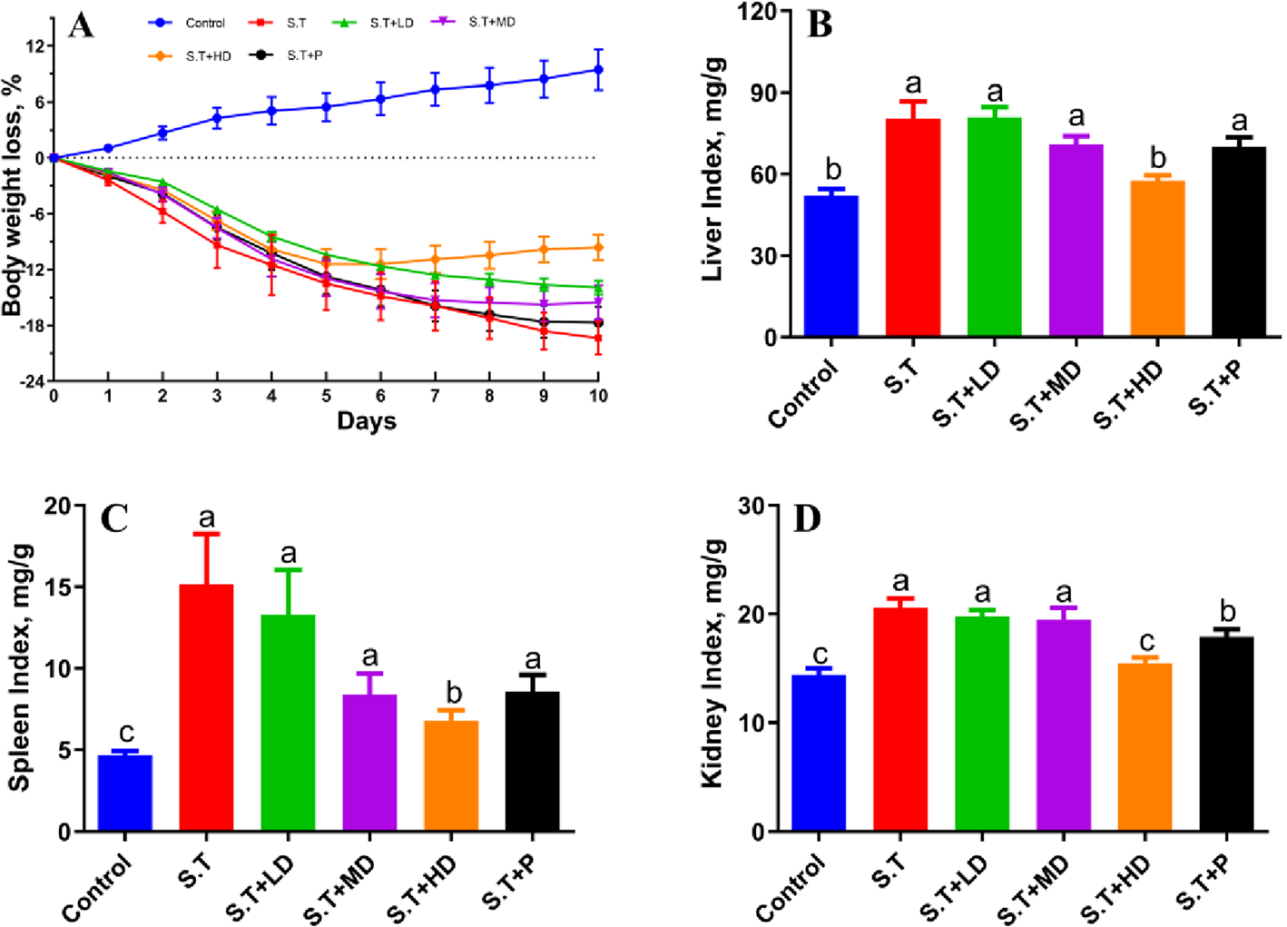
Effects of EPSs on the mouse body weight and organ indices. **A** Changes in the body weight; **B** Liver index; **C** Spleen index; **D** Kidney index. The data are presented as the mean ± SD, *n = 9*. Different letters indicate that the difference between the groups is statistically significant (*P <* 0.05)

The body weight after oral administration of *S.T* was significantly lower than that of the control group (Fig. 2A), and the *S.T*-infected mice exhibited symptoms of decreased appetite, somnolence, diarrhea and flagging spirit, whereas the PBS-treated mice showed no pathology. Oral administration of EPSs and penicillin improved the weight loss of the mice. As shown in Fig. 2A, the body weight of the EPS-treated mice increased, and except the mice in the *S.T* + HD group gained the most weight among all the groups with the exception of the control group. The reduction in body weight observed among the mice in the *S.T* + P group started to be relieved on the ninth day. Oral administration of EPSs and penicillin improved the diarrhea of mice, gradually improved the mental state, and gradually brightened the coat, and the best effect was observed in the *S.T* + HD group. As shown in Fig 2B, C and D, the organ indices of the liver, spleen and kidney of the *S.T* group were significantly higher than those of the control group (*P* < 0.05). No significant difference in the organ indices was found between the *S.T* + LD, *S.T* + MD and *S.T* + P groups and the *S.T* group, whereas the organ indices of the *S.T* + HD group were significantly lower than those of the *S.T* group (*P* < 0.05).

### EPSs reversed the serum cytokine levels of *S.T*-infected mice

**Fig. 3:**
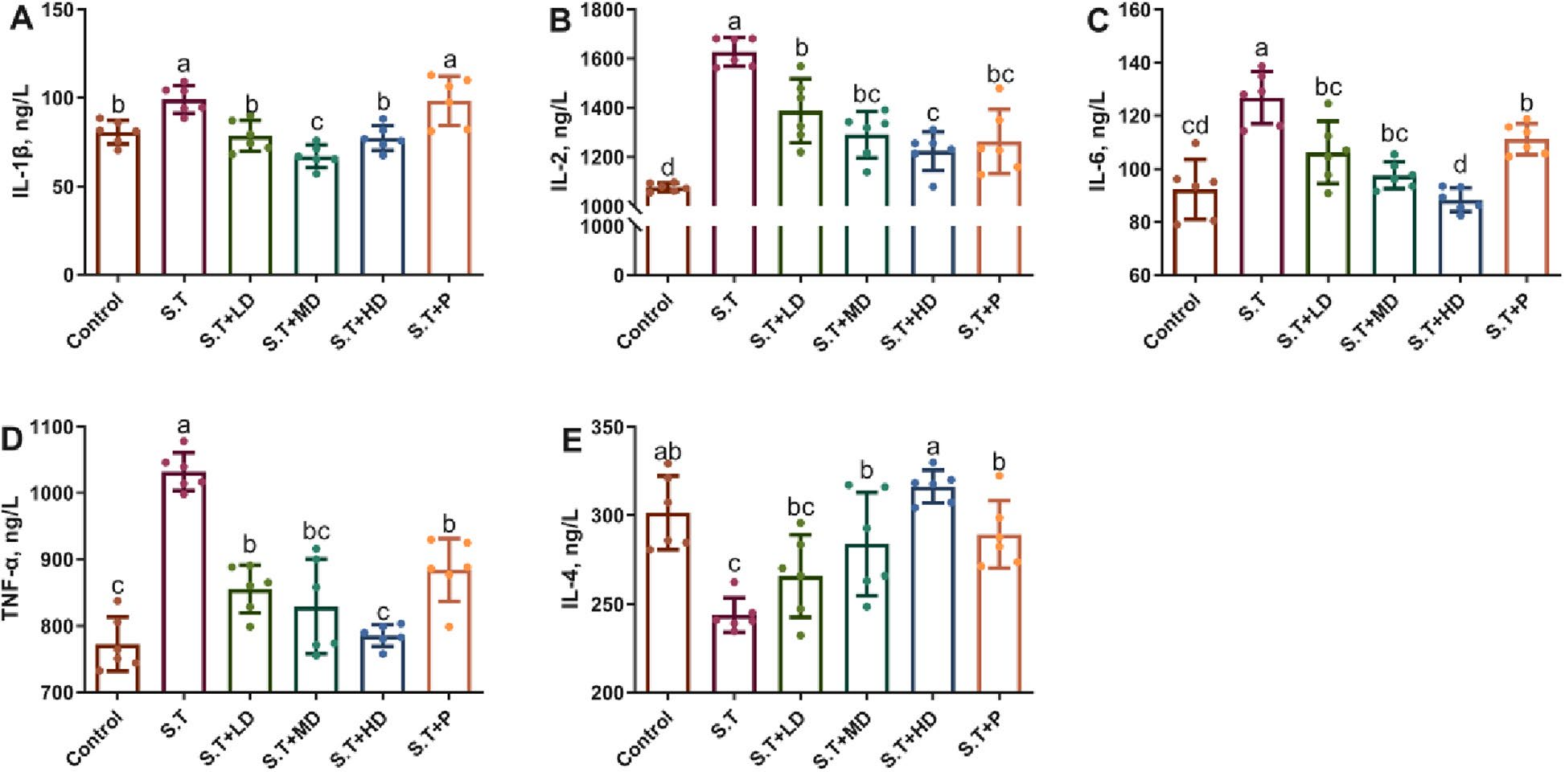
Effects of EPSs on serum inflammatory cytokines in *S.T*-infected mice. The (**A**) IL-1β, (**B**) IL-2, (**C**) IL-6, (**D**) TNF-α and (**E**) IL-4 levels were measured by ELISA. The data are presented as the mean ± SD, *n* = 6. Different letters indicate that the difference between the groups is statistically significant (*P* < 0.05)

The cytokine levels in mouse serum were determined by ELISA. As shown in Fig. 3, *S.T* infection significantly increased the IL-1β, IL-2, IL-6 and TNF-α levels compared with those in the control group (*P* < 0.05). Compared with those of the control group, oral administration of EPSs for 7 d significantly decreased the levels of IL-1β, IL-2, IL-6 and TNF-α (*P* < 0.05), and oral penicillin reduced the levels of IL-2, IL-6 and TNF-α (*P* < 0.05). Moreover, treatment with EPSs and penicillin reversed the *S.T*-induced reduction in the expression level of IL-4 (*P* < 0.05), and no significant difference was found between these treatments and the control group (*P* > 0.05).

### EPSs ameliorated the *S.T*-induced histological damage in the mouse ileum

**Fig. 4:**
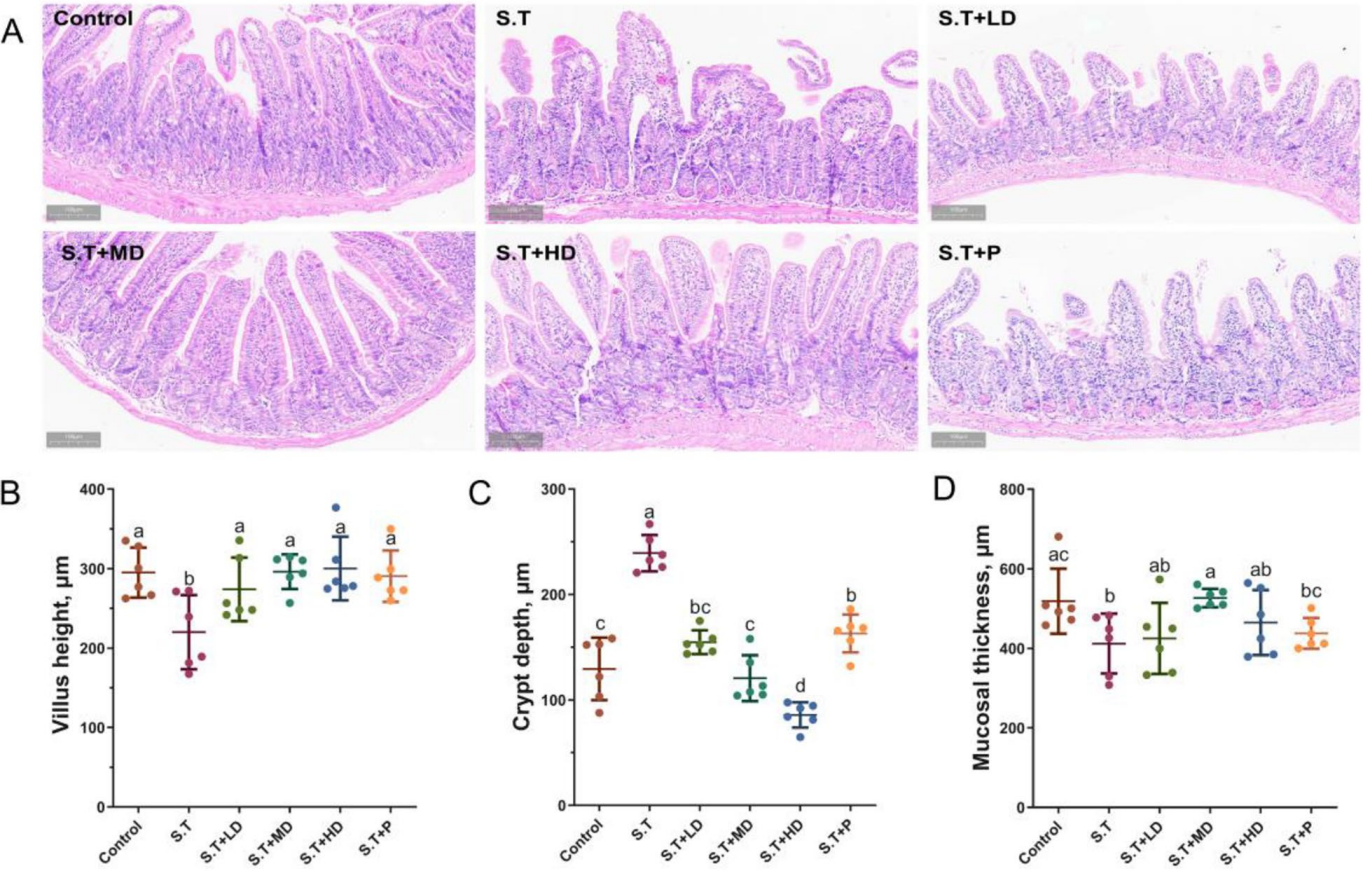
Effects of EPSs on the ileal morphology. **A** Histological change; **B** Villus height; **C** Crypt depth and **D** Mucosal thickness. The data are presented as the mean ± SD, *n* = 6. Different letters indicate that the difference between the groups is statistically significant (*P < *0.05)

Histopathology examination is considered a standard method for assessing the degree of injury in tissues. As shown in Fig. 4A, *S.T* infection disrupted the integrity of mouse ileal villi by causing them to shrink, break and even disintegrate. However, groups with *S.T* + MD, *S.T* + HD and *S.T* + P could ameliorate *S.T*-induced histological damage to the ileum. Measurements of the villus height, crypt depth and mucosal thickness were performed to further evaluate the degree of ileum injury. The results in Fig. 4B, C and D show that oral administration of *S.T* caused significant decreases in the villus height and mucosal thickness (Fig. 4B, D) and an increase in the crypt depth (Fig. 4C) in the ileum compared with the control group (*P* < 0.05). No significant difference in the villus height was observed between the control group and the treatment groups (Fig. 4B). The crypt depth of the *S.T* + LD, *S.T* + MD, *S.T* + HD and *S.T* + P groups was significantly lower than that of the *S.T* group (*P* < 0.05). Although the crypt depth of the *S.T* + HD group was significantly lower than the control group (*P* < 0.05), no significant difference was found among the *S.T* + LD, *S.T* + MD and control groups (*P* > 0.05). As shown in Fig. 4D, no significant difference in the mucosal thickness was found among the *S.T* + LD, *S.T* + HD and *S.T* + P groups (*P* > 0.05), and the mucosal thickness of the *S.T* + MD group significantly greater than that of the *S.T* group (*P* < 0.05).

**Fig. 5:**
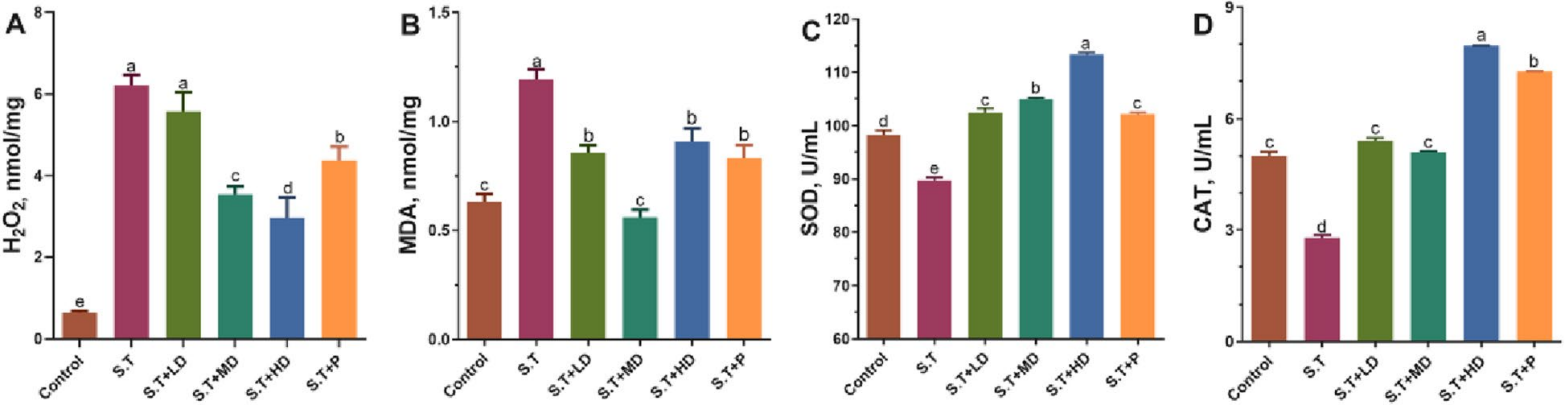
Effects of EPSs on intestinal antioxidant enzyme activity in *S.T*-infected mice. The levels of (**A**) H_2_O_2_, (**B**) MDA, (**C**) SOD and (**D**) CAT in the ileum of mice were measured. The data are presented as the mean ± SD, *n* = 3. Different letters indicate that the difference between the groups is statistically significant (*P < *0.05)

### EPSs alleviated ileal oxidative damage induced by *S.T*

The effect of EPSs on the activity of antioxidant enzymes in the ileum of mice infected with *S.T* is shown in Fig. 5. The *S.T* group exhibited significantly higher levels of H_2_O_2_ and MDA (*P* < 0.05) and significantly lower levels of SOD and CAT (*P* < 0.05) compared with the control group. In addition, the *S.T* + LD and *S.T* + MD groups showed significantly lower levels of H_2_O_2_ and MDA (*P* < 0.05) and significantly higher levels of SOD and CAT (*P* < 0.05) compared with the *S.T* group. The *S.T* + HD group exhibited significantly lower levels of H_2_O_2_ (*P* < 0.05) and significantly higher levels of SOD and CAT (*P* < 0.05) compared with the *S.T* + P group.

### EPSs reversed the *S.T*-induced increases in the levels of ileal inflammatory cytokines

**Fig. 6:**
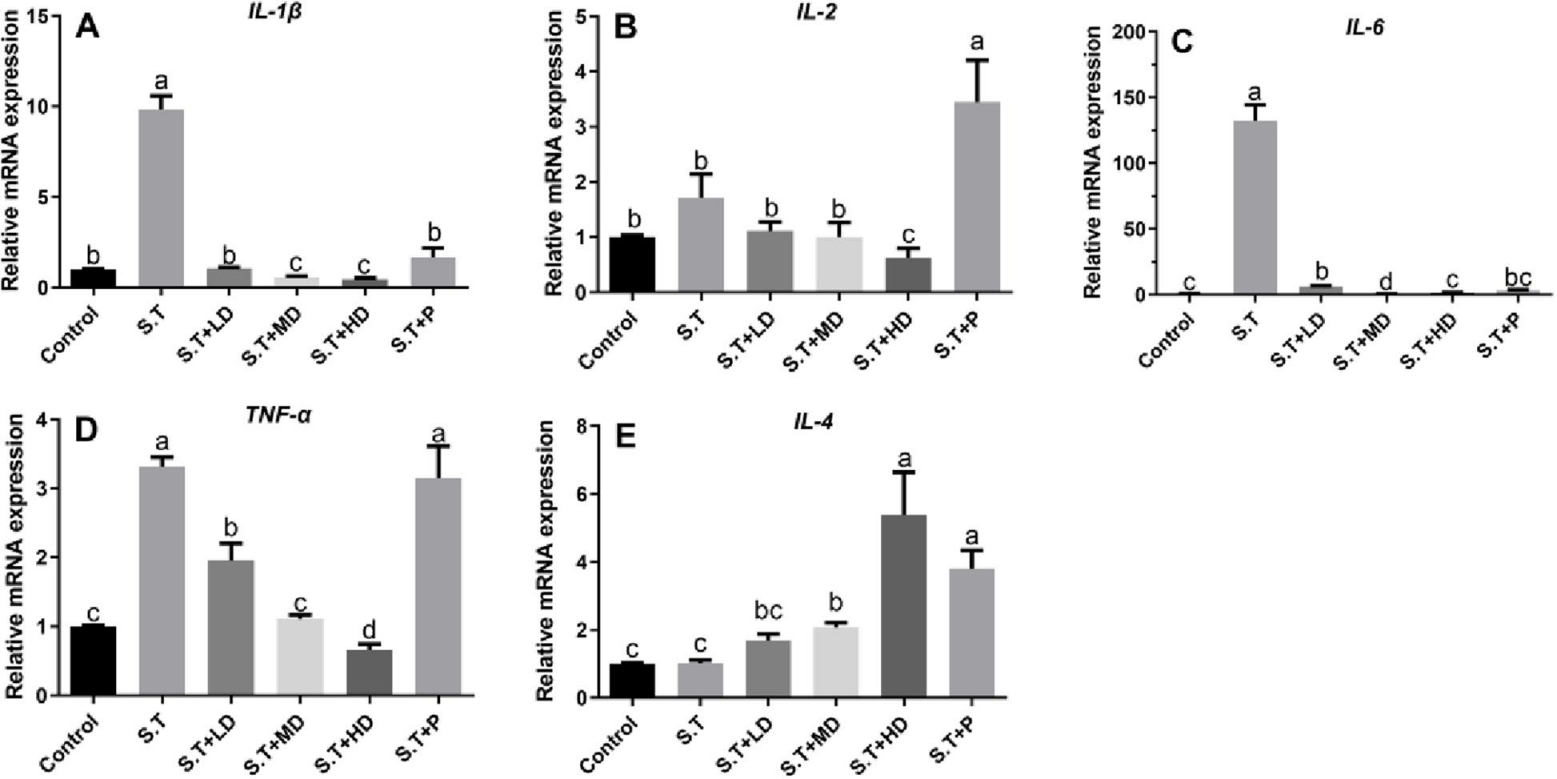
Effects of EPSs on inflammatory cytokines in *S.T*-infected mice. The (**A**) *IL-1β*, (**B**) *IL-2*, (**C**) *IL-6*, (**D**) *TNF-α* and (**E**) *IL-4* levels after oral administration of *S.T* with and without treatment with EPSs and penicillin were measured. The data are presented as the mean ± SD, *n* = 4. Different letters indicate that the difference between the groups is statistically significant (*P < *0.05)

The measurement of the mRNA levels of inflammatory cytokines in the ileum of mice showed that the oral administration of *S.T* increased the expression levels of *IL-1β*, *IL-2*, *IL-6* and *TNF-α* (*P* < 0.05) compared with those of the control group (Fig. 6A, B, C and D), and no significant difference in the expression level of *IL-4* was found between the *S.T* group and the control group (Fig. 6E). Compared with the *S.T* group, treatment with EPSs and penicillin significantly decreased the expression levels of *IL-1β*, *IL-2*, *IL-6* and *TNF-α* and significantly increased the expression levels of *IL-4* (*P* < 0.05) (Fig. 6E). The *S.T* + P group exhibited a significantly higher expression level of *IL-2* and significantly lower expression levels of *IL-1β* and *IL-6* compared with the *S.T* group (Fig. 6A, B, C, E).

### EPSs reduced immune responses through the TLR4/NF-κB/MAPK pathway

**Fig. 7:**
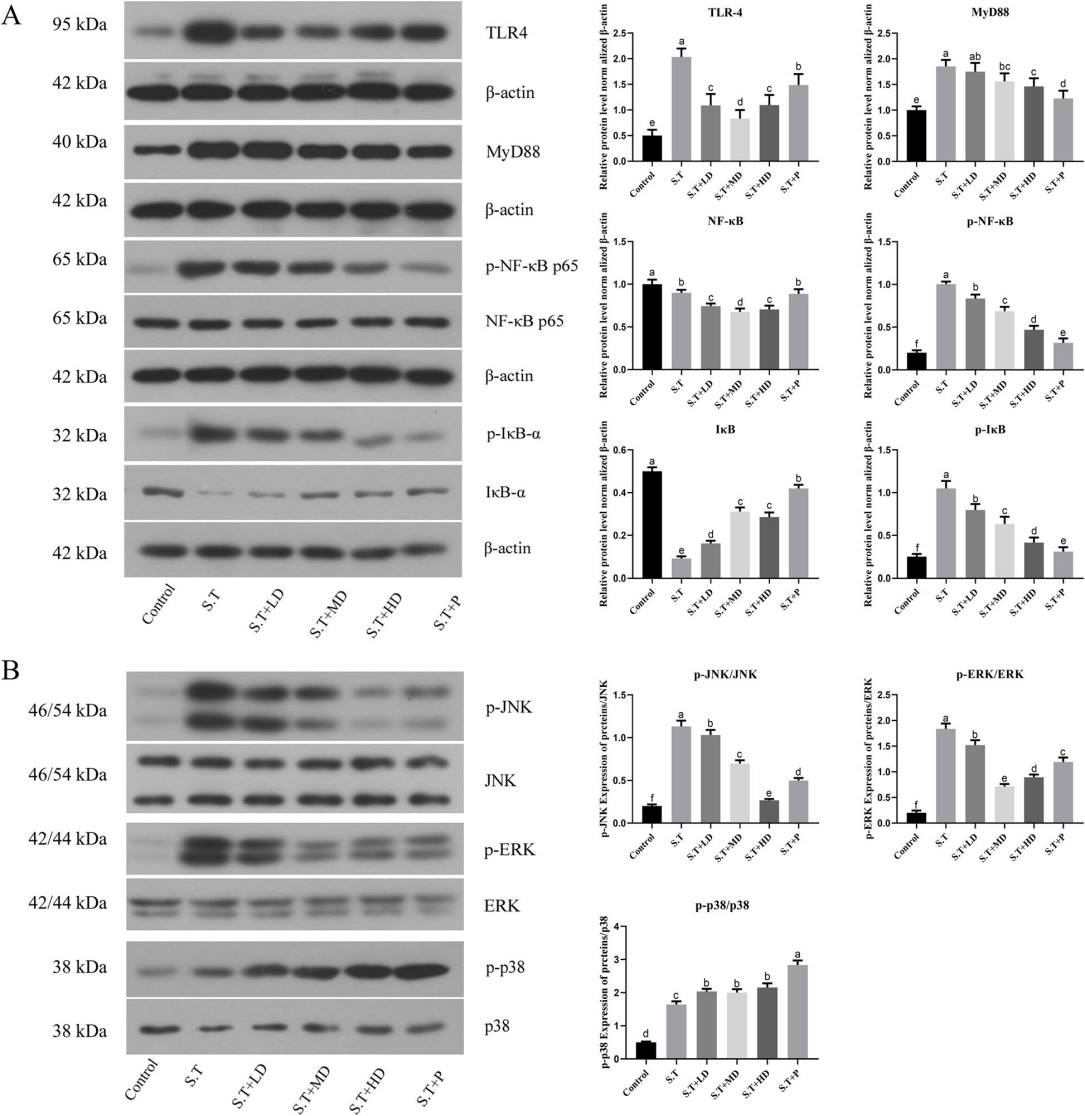
**A** Effects of EPSs on the nuclear translocation of NF-κB and the protein expression of NF-κB-dependent proteins in the *S.T*-induced mouse intestine. **B** EPSs inhibit the *S.T*-induced activation of key proteins of the MAPK pathway. The data are presented as the mean ± SD, *n* = 3. Different letters indicate that the difference between the groups is statistically significant (*P < *0.05)

The above-described findings suggest that EPSs could alleviate the elevated levels of inflammatory factors caused by bacterial invasion. To further investigate the molecular mechanism of EPSs, we conducted the following Western blot experiment. As shown in Fig. 7A, the expression levels of TLR4 and MyD88 proteins were significantly increased in the ileum of mice infected with *S.T* (*P* < 0.05), and these effects were effectively alleviated by both EPSs and penicillin. Moreover, EPSs and penicillin significantly inhibited the *S.T*-induced p65 nuclear translocation and the *S.T*-induced phosphorylation and degradation of IκB in ileal tissue. The results shown in Fig. 7B suggest that the phosphorylation levels of p38, ERK and JNK were significant higher in the *S.T* group compared with the control group (*P* < 0.05). In addition, treatment with EPSs and penicillin significantly inhibited the phosphorylation of ERK and JNK (*P* < 0.05). These results suggest that EPSs could inhibit the expression and activation of key proteins of the TLR4/NF-κB/MAPK pathway and thereby suppress the level of *S.T*-induced ileal inflammation.

### EPSs had no direct anti-*S.T* activity

**Fig. 8:**
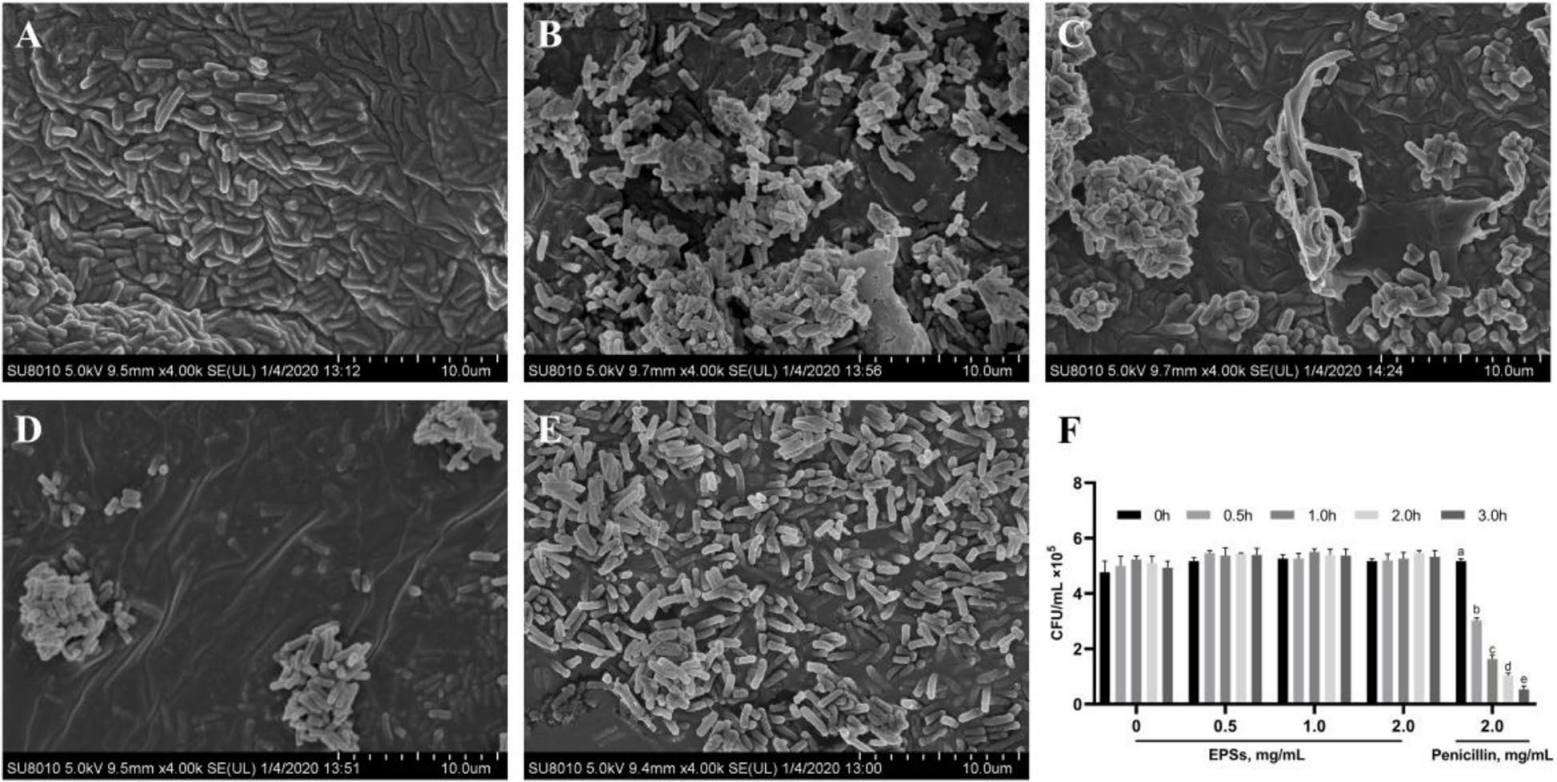
Antibacterial activity of EPSs. SEM micrographs of **A** control cells, **B** 0.5 mg/mL EPSs-treated *S.T* cells, **C** 1.0 mg/mL EPSs-treated *S.T* cells, **D** 2.0 mg/mL EPSs-treated *S.T* cells, **E** 2.0 mg/mL penicillin-treated *S.T* cells. **F** Analysis of the bactericidal kinetics of EPSs. The data are presented as the mean ± SD, *n* = 3. Different letters indicate that the difference between the groups is statistically significant (*P < *0.05)

**Fig. 9:**
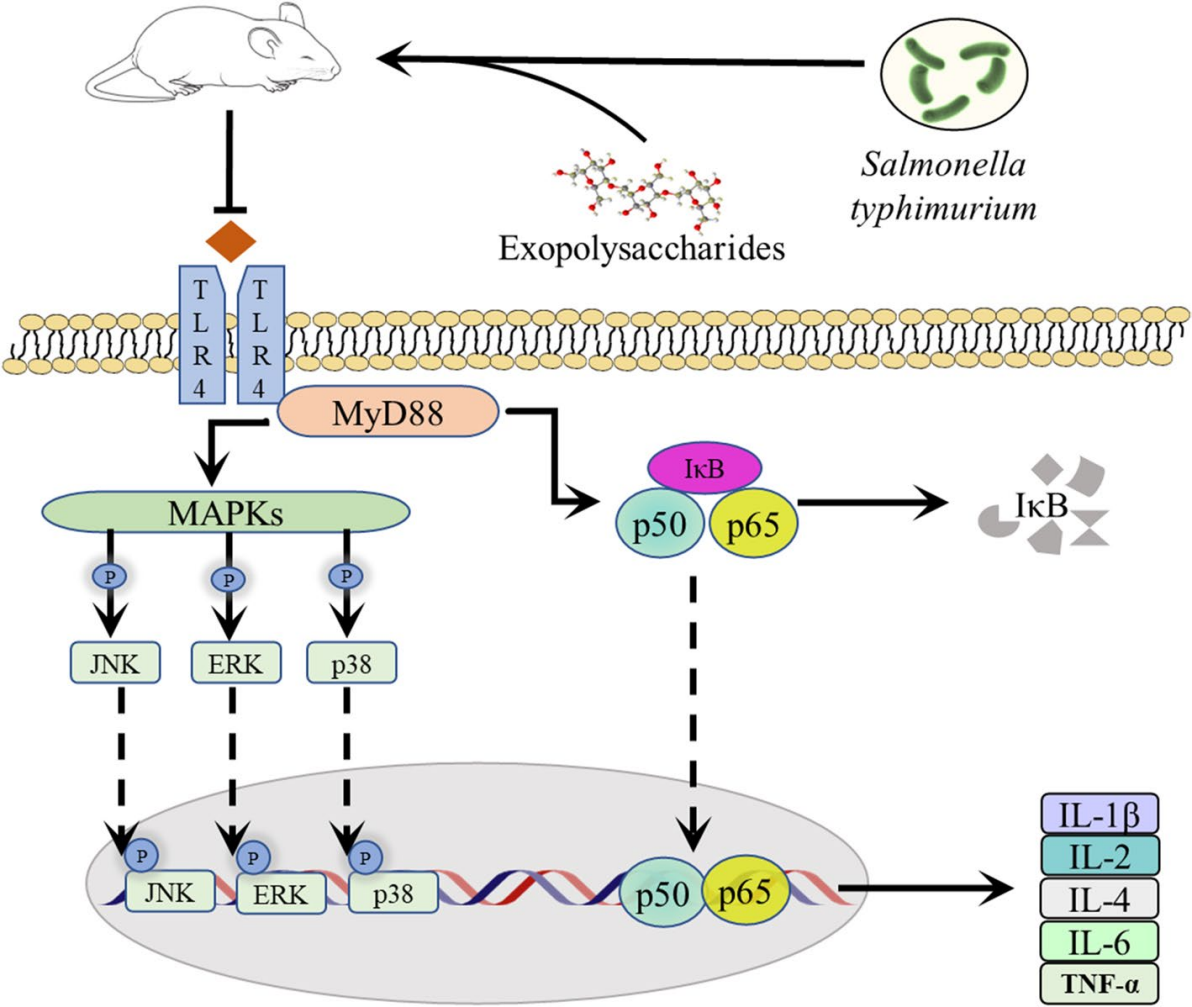
Diagram showing the mechanism through which EPSs attenuate intestinal inflammation through the TLR4/NF-κB/MAPK pathway

To further explore the interaction between EPSs and *S.T*, the morphological structure of EPSs and penicillin-treated *S.T* was observed by SEM*.* As shown in Fig. 8, EPSs-treated *S.T* cells, similar to untreated *S.T* cells, showed a complete membrane surface, but the results clearly show that EPSs promote bacterial agglutination, whereas untreated bacteria were evenly distributed across the field of vision. Penicillin-treated *S.T* cells showed cell membrane wrinkling as well as partial rupture. The analysis of the killing bacterial kinetics revealed that EPSs had no directed anti-*S.T* activity (Fig. 8F) and that penicillin exerted a good bactericidal effect. Thus, EPSs of LGG alleviated *S.T*-induced intestinal inflammation not by killing bacteria but by modulating intestinal immune responses (Fig. 9).

## Discussion

*S.T* infection can influence various intestinal barrier dysfunctions, including histological destruction, oxidative damage, and immune responses of the intestinal tract, and these effects result in gastric oxidative stress, hemorrhagic ulcers and even various organ diseases [2, 19, 20]. In our study, oral administration of *S.T* caused significant weight loss, organ enlargement and atrophy and rupture of ileal villi in mice, which manifested as decreases in the villus height and mucosal thickness and an increase in the crypt depth. These phenomenon indicated that *S.T* led to an increase in the intestinal permeability and intestinal barrier dysfunction and ultimately led to anorexia, weight loss, and inflammation. Previous colonization of hysterectomy-derived preterm gnotobiotic piglets with LGG protected against subsequent infection with *S.T*, including ameliorating histopathological changes in the intestine and reducing the IL-8 levels in the jejunum and plasma and the IL-12/23 p40 level in the jejunum [21]. Our previous study found that EPSs from LGG could alleviate the oxidative damage and apoptosis of IPEC-J2 cells caused by H_2_O_2_ [18]. However, the effects of EPSs of LGG against bacteria-induced intestinal damage remain unknown. Our study showed that oral administration of EPSs of LGG alleviated *S.T*-induced intestinal morphological damage and reduced the organ indices of the liver, spleen and kidney. Notably, the highest tested concentration of EPSs (2.0 mg/mL) also increased the body weight of mice appropriately compared with those of the other treatment groups.

Host innate and adaptive immune responses are stimulated when the bacteria arrive and colonize the intestine. Harmful conditions induce the production of reactive oxygen species (ROS) [22], which is a major cause of intestinal oxidative stress and one of the main factors causing intestinal diseases, such as inflammatory bowel disease, intestinal mucosal infection, Crohn’s disease, ulcerative colitis, and colon cancer [23, 24]. CAT and SOD are important antioxidant biomarkers, whereas MDA is an important oxidative biomarker. SOD converts O_2_^−^ into H_2_O_2_ and O_2_, and CAT is involved in the process of scavenging H_2_O_2_ to H_2_O [25]. In contrast, ROS, such as H_2_O_2_ caused by bacterial infection, result in endogenous lipid peroxidation, which produces MDA, a biomarker of lipid degradation [26]. The invasion of *S.T* reduced the levels of SOD and CAT and increased the levels of H_2_O_2_ and MDA in the ileum. According to a previous study, EPSs of LGG extracted by ethanol precipitation could effectively eliminate most oxygen free radicals, indicating that EPSs effective antioxidant activity in vitro [18]. Here, we administered EPSs orally to *S.T-*infected mice and measured the activity of antioxidant enzymes in ileal tissues. EPSs of LGG also exerted a considerable antioxidant effect in vivo. In *S.T-*infected mice, oral treatment with EPSs for 7 d increased the activities of SOD and CAT and decreased the accumulation of H_2_O_2_ and MDA.

As expected, the oral administration of *S.T* induced an immune response in the ileum and even spreading throughout the body. Decreased and increased expression levels of inflammatory factors are a signal of a reduction of neutrophil migration to improve the development of intestinal inflammation [27]. Among these factors, TNF-α is a key regulator of the inflammatory response, and its level is correlated with the severity of infection and inflammatory response in serum and tissues [28]. IL-1β, a proinflammatory cytokine released by various immune modulating cells, is related to intestinal inflammation [29]. Increases in the levels of TNF-α and IL-1β can stimulate the secretion of the potent proinflammatory cytokine IL-6 by intestinal epithelial cells [30]. IL-4, a pleiotropic anti-inflammatory cytokine, mediates the profile of cytokine production of CD4^+^ helper T cells toward a Th2 paradigm cytokine response, which mainly functions by suppressing the proinflammatory milieu [31]. IL-2 is a potent T-lymphocyte growth factor produced by CD4^+^ lymphocytes and plays a pivotal role in the immune response [32]. Numerous studies have shown that TNF-α, IL-1β and IL-4 can influence the expression of tight junction proteins and cause an increase in intestinal epithelial permeability, whereas IL-6 exerted a barrier protective effect via upregulation of Keratin 8 and 18 to reduce the incidence of bacterial translocation [33, 34]. Our study showed that the invasion of *S.T* induced overexpression of *IL-1β*, *IL-2*, *IL-6* and *TNF-α* and inhibited the production of *IL-4* in the ileum. Treatment with EPSs inhibited the production of cytokines that promote inflammation and upregulated the production of cytokines that suppress inflammation. Therefore, we hypothesized that EPSs may regulate intestinal immune cell secretion of cytokines by maintaining the intestinal barrier function and improving the intestinal integrity, and these effects ultimately alleviate *S.T*-induced intestinal injury in mice. Moreover, the redox balance is responsible for the initiation and progression of inflammation [35], and oral EPSs maintain the redox balance in ileal tissue, which is an important reason for why EPSs maintain intestinal health.

To further elucidate the protective mechanism of EPSs in the *S.T*-induced intestinal bacterial infection model, Western blot assays were conducted to explore the molecular mechanisms of the anti-inflammatory role of EPSs. When bacteria invade the intestine, gastrointestinal epithelial cells provide a natural barrier. Pattern-recognition receptors serve as the first barrier against pathogen invasion, and among them, the Toll-like receptor (TLR) is one of the main receptors and acts as a transmembrane receptor participating in signal transmission [36]. TLR4, an upstream receptor of the MAPK/NF-κB pathway, can combine with Gram-negative bacterial extracellular lipopolysaccharide by identifying cognate ligands [37]. TLR4 recruits its downstream adaptor MyD88 via interactions with the Toll-interleukin-1 receptor domains to activate downstream NF-κB signaling pathways [37]. As shown in Fig. 7A, *S.T* invasion increased the protein expression levels of TLR4 and MyD88, and the oral administration of EPSs and penicillin weakened this trend, demonstrating that *S.T* invasion activates the NF-κB signaling pathway to induce subsequent inflammatory responses. The NF-κB signaling pathway is an important regulatory factor involved in immune responses, cell growth and the expression of proliferation and apoptosis genes, which play pivotal roles in disease prevention and development. Under normal physiological conditions, proteins are bound and inhibited by IκB proteins and are inactive in the cytoplasm [38]. When exposed to exogenous stimuli, such as bacteria, NF-κB is activated and transferred to the nucleus via phosphorylation of IκB, which stimulates the expression of inflammatory cytokines and results in inflammatory responses [39]. In this study, the oral administration of EPSs and penicillin inhibited *S.T*-induced NF-κB p65 nuclear translocation and IκB protein phosphorylation. The above-described results demonstrated that EPSs could inhibit the expression of key proteins in the TLR4/NF-κB pathway to exhibit an inhibitory effect against *S.T*-induced ileal inflammation.

Furthermore, the activation of TLR4 triggers the MAPK signaling pathway, leading to the production of proinflammatory factors [40]. ERK, JNK and p38 are 3 subfamilies of MAPK. These proteins can accelerate cell proliferation and growth, and activation of p38 and JNK accelerates apoptosis [41]. The phosphorylation of p38/MAPK, ERK/MAPK and JNK/MAPK is associated with the induction of inflammatory factors via the MAPK signaling pathway, including TNF-α and IL-6 [42]. In this study, *S.T* invasion increased the phosphorylation of JNK, ERK and p38, and treatment with EPSs and penicillin inhibited the *S.T*-induced phosphorylation of JNK and ERK but not p38. These results illustrated that EPSs inhibit inflammatory responses by blocking the MAPK signaling pathway, and the reason for why the phosphorylation level of p38 increased after EPSs treatment should be further studied in the future.

It has been proposed that EPSs have antibacterial activity either directly by disrupting bacterial structure or indirectly by modulating the release of immune factors release from immune organs or immune cells [43]. EPSs produced by *Lactobacillus reuteri* SHA101 and *Lactobacillus vaginalis* SHA110 were found to have direct antibacterial activity against *S.T* in vitro; a concentration of the corresponding EPSs of 4.0 mg/mL resulted in inhibition zone diameters of 14.0 ± 1.5 mm and 15.0 ± 0.6 mm, respectively, which significantly inhibited the growth of *S.T* [43]. In this study, we discovered that EPSs of LGG did not directly inhibit or kill *S.T* in vitro and that EPSs could not destroy the structure of *S.T*, but could induce the aggregation of *S.T*, and a higher concentration of EPSs resulted in a higher degree of bacterial aggregation. It has been reported that EPSs promote phagocytosis of bacterial clusters by phagocytes and inhibit their invasion of intestinal epithelial cells [44]. In this study, it is assumed that EPSs may exert antibacterial activity by modulating immune organs or immune cells, releasing cytokines and promoting phagocytosis of bacterial clusters by phagocytes. Scanning electron microscopy was used to examine the interaction between penicillin and *S.T*, and the results showed that penicillin could cause wrinkling and even rupture of *S.T* membranes, which confirmed that penicillin could directly inhibit *S.T* growth.

## Conclusions

In summary, EPSs of LGG were able to exhibit good antioxidant activity in vivo and alleviated *S.T*-induced intestinal injury in mice by regulating the TLR4/NF-κB/MAPK pathway. This study constituted the first exploration of the therapeutic effect and mechanism of EPSs from LGG on diarrhea induced by bacterial infection in mice. Because the production of bacterial EPSs is too low and bacterial EPSs cannot be synthesized artificially, this study provides a reference for the application of bacterial EPSs in other animals and humans and provides evidence showing that EPSs could be an effective alternative to antibiotics.

## Data Availability

All the data generated or analyzed during the present study are available from the corresponding author on reasonable request.

## Abbreviations

CAT: Catalase
EPSs: Exopolysaccharides
H&E: Hematoxylin and eosin
H_2_O_2_: Hydrogen peroxide
IL: Interleukin
IPEC-J2: Intestinal porcine epithelial
LB: Luria-Bertani
LGG: *Lactobacillus rhamnosus*
MDA: Malondialdehyde
MHA: Mueller Hinton agar
PVDF: Polyvinylidene fluoride
ROS: Reactive oxygen species
SEM: Scanning electron microscopy
SOD: Superoxide dismutase
*S.T*: *Salmonella typhimurium*
TBST: Tween 20/Tris buffered saline
TNF-α: Tumor necrosis factor-α
TLR: Toll-like receptor
